# Action potential responses to changes in stimulation frequency and isoproterenol in rat ventricular myocytes

**DOI:** 10.14814/phy2.15166

**Published:** 2022-01-25

**Authors:** Luke A. Howlett, Hannah M. Kirton, Moza M. Al‐Owais, Derek Steele, Matthew K. Lancaster

**Affiliations:** ^1^ Faculty of Biological Sciences University of Leeds Leeds UK

**Keywords:** action potential, adrenergic response, cardiovascular, Electrophysiology, exercise

## Abstract

**Purpose:**

Current understanding of ventricular action potential adaptation to physiological stress is generally based on protocols using non‐physiological rates and conditions isolating rate effects from escalating adrenergic stimulation. To permit refined understanding, ventricular action potentials were assessed across physiological pacing frequencies in the presence and absence of adrenergic stimuli. Isolated and combined effects were analyzed to assess their ability to replicate in‐vivo responses.

**Methods:**

Steady‐state action potentials from ventricular myocytes isolated from male Wistar rats (3 months; N = 8 animals) were recorded at 37°C with steady‐state pacing at 1, 2, 4, 6, 8 and 10 Hz using whole‐cell patch‐clamp. Action potential repolarization to 25, 50, 75, 90 and 100% of full repolarization (APD_25‐100_) was compared before and after 5 nM, 100 nM and 1 µM isoproterenol doses.

**Results:**

A Repeated measures ANOVA found APD_50‐90_ shortened with 5 nM isoproterenol infusion by 6–25% (but comparable across doses) (*p* ≤ 0.03). Pacing frequencies emulating a normal rat heart rate (6 Hz) prolonged APD_50_ 23% compared with 1 Hz pacing. Frequencies emulating exercise or stress (10 Hz) shortened APD_90_ (29%).

**Conclusion:**

These results demonstrate modest action potential shortening in response to adrenergic stimulation and elevations in pacing beyond physiological resting rates. Our findings indicate changes in action potential plateau and late repolarization predominantly underlie simulated exercise responses in the rat heart. This work provides novel action potential reference data and will help model cardiac responses to physiological stimuli in the rat heart via computational techniques.

**Highlights**

• Our findings demonstrate action potential duration (AP_50‐90_) shortens by 16–28% with increments in pacing above the typical resting heart rate of the rat towards rates reflecting heavy and maximal exercise (6 vs 8 and 10 Hz).
• Action potential duration (AP_50‐90_) shortens by an average of 6–25% at each frequency with submaximal (5 nM), and 5–31% (on average) with near maximal (100 nM) adrenergic stimulation, though responses to maximal adrenergic stimulation by isoproterenol (1 μM) were more heterogeneous than those seen with lower doses (responses ranging from 18% prolongation to 20% shortening).
• Action potentials showed a trend of dampened sensitivity to adrenergic stimulation when myocytes were paced at physiological frequencies compared to the response seen during commonly used 1 Hz pacing.
• This work provides novel reference data demonstrating the range of ventricular action potential adaptation under simulated physiological conditions.

## 1 INTRODUCTION

Tight control of cardiac electrical activity is fundamental to maintaining a healthy cardiac output and function at rest and in response to stress. Normal electrical activity relies on coordinated generation and propagation of action potentials (APs) across the myocardium which respond rapidly and systemically to neurohormonal changes and thus provide the cellular excitation required to facilitate coupling to contractile processes and produce cohesive cardiac contractions (Bers, 2002; Grant, 2009; Pinnell et al., 2007). Analysis of the cardiac AP provides information on the normal electrical adaptation to stress but can also identify pathophysiological responses potentially associated with arrhythmias and dysfunction (Feridooni et al., 2015; Gan et al., 2013; Herraiz et al., 2013; Li et al., 2004; Liu et al., 2000; Ocorr et al., 2007; Sorrentino et al., 2016).

The cardiac AP reacts to increased adrenergic drive as a result of stress or exercise (Grant, 2009). During exercise, sympathetic activation is increased, leading to increased circulation of adrenaline and noradrenaline (Ferrara et al., 2014; Gordan et al., 2015; Howlett & Lancaster, 2021; McCorry, 2007). These hormones predominantly bind to the beta_1_ adrenergic receptor (β_1_AR), triggering a cascade of downstream signaling via the adenyl cyclase (AC)/ cyclic adenosine monophosphate (cAMP)/ protein kinase A (PKA) pathway (Fink et al., 2001; Howlett & Lancaster, 2021; Jeevaratnam et al., 2018; Layland et al., 2005; Pare et al., 2005; Rochais et al., 2004; Sampson & Kass, 2010; Saucerman & McCULLOCH, 2006). Action potential repolarization is heavily modulated by β_1_AR signaling, where numerous changes in ion flux are documented to give rise to significant shortening of the AP duration (APD) (although less‐often prolongation has also been documented) (Kang et al., 2017; Stuart et al., 2018; Wang & Fitts, 2017, 2020). This leads to the changes in cardiomyocyte inotropy, chronotropy and lusitropy that enable cardiac output to dynamically vary to cope with the demands of exercise.

During experimental investigation of cardiomyocytes, activation frequency is typically controlled to maintain a stable activation/ pacing rate. This activation frequency can be adapted to reproduce a range of beating frequencies. However, many investigations utilize low activation frequencies relative to the typical physiological range of the animal models used (Banyasz et al., 2014; Bao et al., 2013; Cerbai et al., 1994; Farrell & Howlett, 2007; Gan et al., 2013; Kang et al., 2017; Liu et al., 2000; Natali et al., 2002; Sala et al., 2017). Activation frequencies of 1 Hz are common, reflecting a heart rate of ~60 bpm (Banyasz et al., 2014; Bao et al., 2013; Kang et al., 2017). Such activation rates may mimic the lower range of the resting human heart rate but are much lower than typical resting heart rates for rodents, which are the most common source of cardiac myocytes used in experimentation. Lower activation frequencies are commonly utilized due to the greater stability seen at such frequencies in single cells, key in electrophysiological experiments, and the subsequent positive impact on repeated patch‐clamp success in what can be a demanding low‐yield experimental technique. Also, the investigation of higher frequencies may be limited by the kinetics of the ion channels particularly when measurements are made at ambient rather than at physiological temperatures. As such, whilst recordings made at room temperature and slow stimulation rates can give information about the general properties of the ion channels present, such conditions do not come close to replicating the normal physiological environment within which the donor heart for the cells would have operated.

Whilst changing activation frequency emulates part of the cardiac response to exercise and stress, it does not recreate the modification of ion channels and ion handling which will occur physiologically due to the associated exposure to adrenergic stimulation. In many experiments this effect has been investigated through the infusion of an adrenergic stimulant at one fixed dose, generally a saturating dose, but this is often done at one stimulation frequency (to control number of variables) and again does not recreate the physiological situation of co‐escalating adrenergic stimuli and activation frequency (Kang et al., 2017; Sala et al., 2017; Stuart et al., 2018; Wang & Fitts, 2017). At present, the influence of adrenergic stimulation on AP repolarization among a range of frequencies, including physiologically relevant frequencies and temperatures in a rat model is not clear. Improving understanding in this area is important for the development of computational models of cardiac function and for greater understanding of AP control under such physiologically relevant conditions.

The aim of this study was to analyze the response of the rat ventricular myocyte AP across a range of activation frequencies with and without increasing doses of isoproterenol in order to mimic key physiological environments of the myocytes responding to stress. Our hypothesis was that as activation rate increases the AP would shorten in a rate‐dependent manner which would be potentiated in a dose‐dependent manner by adrenergic stimulation.

## 2 MATERIALS AND METHODS

### 2.1 Preparation of ventricular myocytes and experimental conditions

A total of eight adult (age 3 months) male Wistar rats (~150–200 g) were housed in the Animal Unit, Faculty of Biological Sciences, University of Leeds. Animals remained in temperature‐controlled rooms, subject to 12‐h light: dark cycles. Food and water were provided ad libitum. Rats were sacrificed by concussion of the brain, followed by cervical dislocation in accordance with Schedule One methods stated in the Animals (Scientific Procedures) Act, 1986 and approved by the University of Leeds ethics committee.

After sacrifice, hearts were rapidly cannulated via the aorta and perfused at 37°C, using retrograde constant flow, with a solution containing (in mM); (Sigma‐Aldrich unless otherwise stated): NaCl 130, KCl 5.4, MgCl 1.4 (Fluka), NaH_2_PO_4_ 0.4 (Acros Organics), HEPES 10, glucose 10, taurine 20, creatine 10, pH 7.3 (NaOH, BDH Laboratory Supplies) to wash all blood from the vascular system. A calcium buffering solution (100 µM EGTA added to the solution) was then perfused for 3 min to prevent beating before starting the dissociation process. Finally, hearts were perfused for 8–10 minutes with a digest solution (50 ml) consisting of: collagenase (50 mg, type 2 Worthington) and protease (5 mg) added to 50 ml of the perfusion solution. All solutions were bubbled with 100% oxygen. Following this, the ventricular tissue was dissected into small strips and shaken (Shaker; Stuart Scientific, Keison Products) in a conical flask at 37°C (temperature‐controlled water bath; Grant Instruments) at a rate of 500–550 oscillations per minute in ~4 ml of enzyme containing solution (with added BSA, 1 mg/ml) for 5 min. Dissociated myocytes were filtered using nylon gauze (200 µm) and centrifuged (500 rpm × 45 – 60 seconds) (Rotina 46R, Hettich Zentrifugen). Supernatant was removed, and the pellet re‐suspended in perfusion solution with 750 µM CaCl. These steps were repeated a maximum of 5 times or until remaining pieces of heart tissue turned pale. Myocytes were stored at room temperature and used within 6–8 h.

### 2.2 Whole‐cell patch‐clamp electrophysiology

Freshly isolated ventricular myocytes were placed in a 2 ml perfusion bath on the platform (Gibraltar, Burleigh) of an upright microscope (UMPlanF1, BX51WI, Olympus), on an anti‐vibration table within a Faraday cage and left to settle for 5 min. Myocytes were perfused with normal Tyrode solution via peristaltic valve assembly (cF‐8VS valve assembly, Cell MicroControls) and flow controller (cFlow Flow Controller, Cell MicroControls) at a rate of 3 ml per minute (Fujisawa et al., 2000). Normal Tyrode solution contained NaCl 136, KCl 4, MgCl 2, CaCl_2_ 1, HEPES 10, glucose 10, pH 7.4 (NaOH). Bath temperature was maintained at 37 ± 1°C (NBD TC2 Bip Temperature Controller, Cell MicroControls). Myocytes were stimulated with borosilicate glass pipettes (1.5 mm O.D × 1.16 mm I.D, Harvard Apparatus) filled with a physiological solution containing KCl 135, EGTA 10, HEPES 10, glucose 5, pH 7.2 (pH adjusted using KOH, Alfa Aesar). Glass pipettes were placed over a silver chloride coated silver wire electrode fitted to the patch‐clamp headstage (CV203BU, Axon Instruments, Molecular Devices). Headstage movement along the x, y and z axes was controlled via a micro‐manipulator (PCS‐6000, Exfo, Burleigh). Electrical activity of myocytes was measured using a low‐noise amp meter (AxoPatch 200B integrating patch clamp, Axon CNS, Molecular Devices). All electrophysiological data was digitized within Clampex (version 10.7) via a data acquisition system (pCLAMP 9.0 software; Digidata 1440A, Axon CNS, Molecular Devices). Low resistance glass pipettes were used for all experiments (4–8 MΩ) (Armstrong & Gilly, 1992). Action potential signals were sampled at 5000 KHz after low‐pass filtering at 1 KHz. Series resistance was compensated for where necessary (≥ 80%) (Armstrong & Gilly, 1992).

All electrophysiology experiments were performed in current‐clamp mode. Depolarizing 2 ms (~ 1.5 nA) current pulses were used to evoke APs. Average access resistance was 19 ± 1.49 MΩ. Average cell capacitance was 136 ± 10.53 pF. No holding current was used. Diastolic membrane potential during control conditions averaged −69.1 mV. After achievement of whole‐cell configuration, steady‐state responses in myocytes were assessed (after pre‐conditioning with stimulation at the relevant frequency for 60 s) at 1, 2, 4, 6, 8 and 10 Hz pacing. Myocytes were then perfused with isoproterenol (5 nM) for 3–5 minutes before AP recordings were repeated (Harding et al., 1988; Johnson et al., 2012; Lim et al., 1999). Myocytes were then perfused with 100 nM isoproterenol for a minimum of 2 min before recording once more. The last isoproterenol dose of 1 μM was perfused for a minimum of 2 min before completing the final recordings.

Isoproterenol doses of 5 nM, 100 nM and 1 μM in this study were used to reflect submaximal, near maximal and supramaximal/saturating doses, emulating, in part, the incrementing adrenergic stimulation in exercise performance. Previous research supports this, with findings indicating an EC_50_ of 1–10 nM isoproterenol regarding impact on AP_90_, whilst 100 nM brings rise to almost a maximal response (Wang & Fitts, 2017).

Individual steady‐state AP recordings were averaged for each separate activation frequency and each separate isoproterenol dose. Time to 25%, 50%, 75%, 90% and 100% repolarization (APD_25_, APD_50_, APD_75_, APD_90_, APD_100_) were measured using the Clampfit software (version 10.7). Diastolic membrane potential, AP amplitude and upstroke velocity were also measured using the same software.

### 2.3 Data analysis

All data presented as mean ± standard error of the mean (SEM). Statistical analyses were completed using IBM statistics 26 software (SPSS Inc., Chicago, IL). A repeated measures analysis of variance (ANOVA) was used to assess differences between variables across activation frequencies and/or isoproterenol doses. Bonferroni post‐hoc analysis was used to assess pair‐wise differences where significant main effects were observed. Statistical significance was set at *p* < 0.05. Data was stored, processed, and organized using Microsoft Excel (2016). All figures were generated using Origin 2018b.

## 3 RESULTS

**FIGURE 1:**
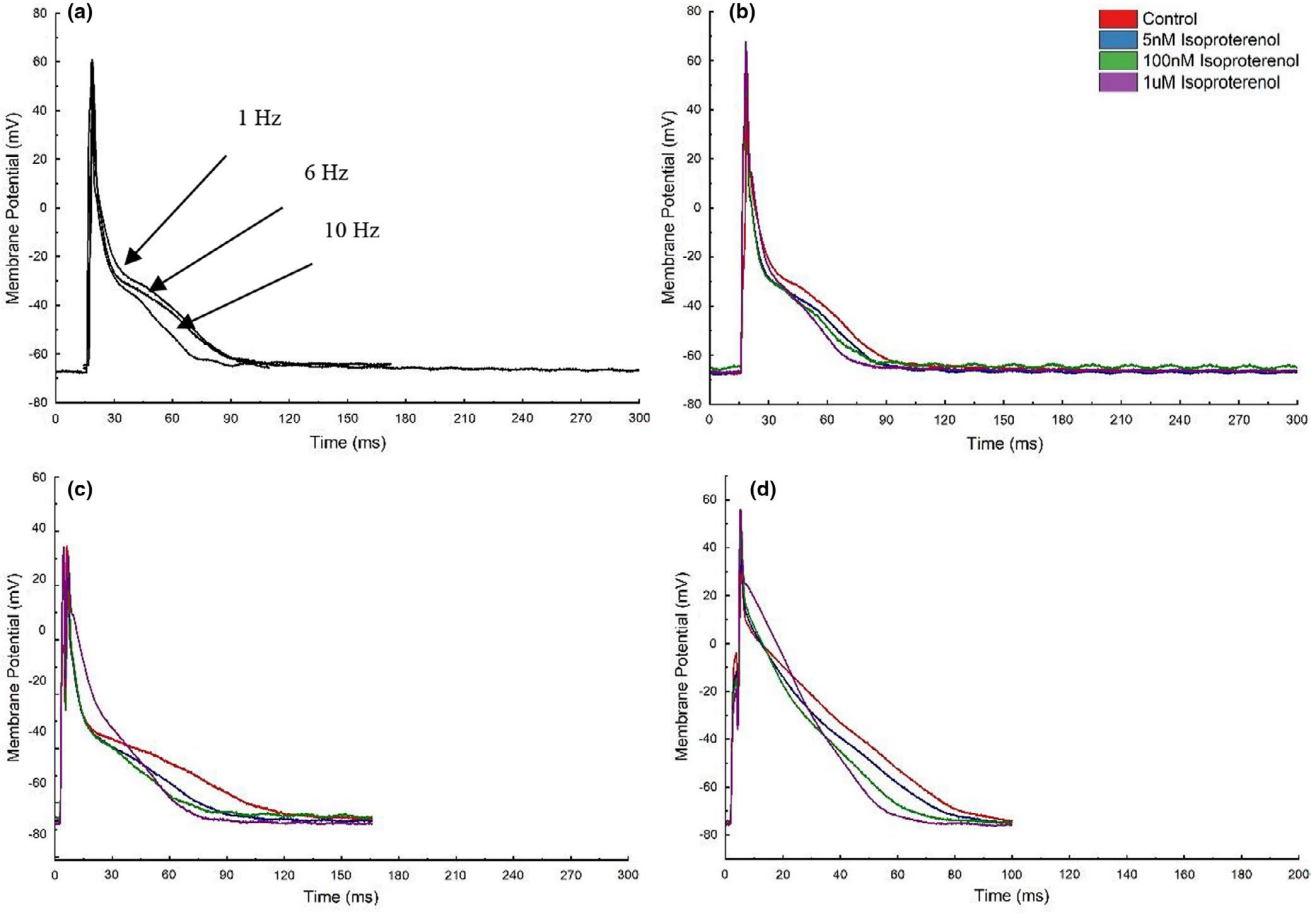
Example overlaid action potentials for a typical ventricular myocyte illustrating the impact of changing activation frequency alone (a) and with incrementing adrenergic stimulation during stimulation at 1 Hz (b), 6 Hz (c) and 10 Hz (d) respectively

A successful set of recordings for all frequencies at all doses of isoproterenol were obtained in 19–22 cells in 8 animals. A typical set of raw recordings are shown in Figure 1. Visually, a shortening of APD was seen in response to the increase in stimulation frequency at pacing frequencies emulating resting and strenuous exercising rat heart rates (6 and 10 Hz) compared to the commonly utilized 1 Hz pacing frequency. Equally Figure 1 displays the observed shortening of APD during the application of isoproterenol. In this example, the combined impact was not obviously additive on initial inspection although isoproterenol visually appears to remodel phases 1 and 2 of the AP in a manner not seen simply with increasing the stimulation frequency.

**FIGURE 2:**
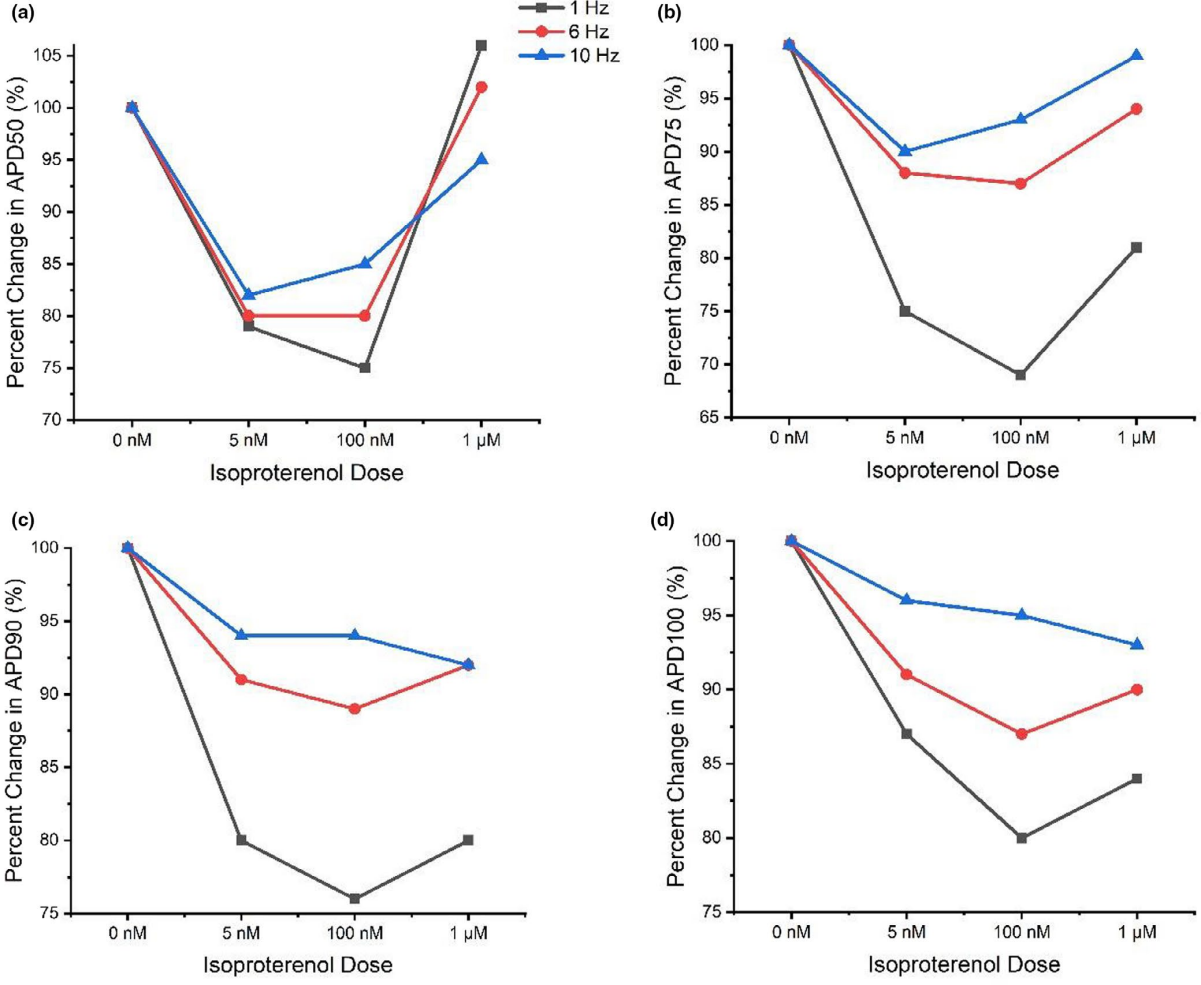
Average isoproterenol responses per animal in APD_50_ (a), APD_75_ (b), APD_90_ (c) and APD_100_ (d) at pacing frequencies typically used in electrophysiology set‐ups (1 Hz) as well as pacing frequencies emulating resting and heavy exercising rat heart rates (6 Hz and 10 Hz). Isoproterenol response data plotted as a percentage of APD_50‐100_ during control conditions (or 0 nM isoproterenol)

**FIGURE 3:**
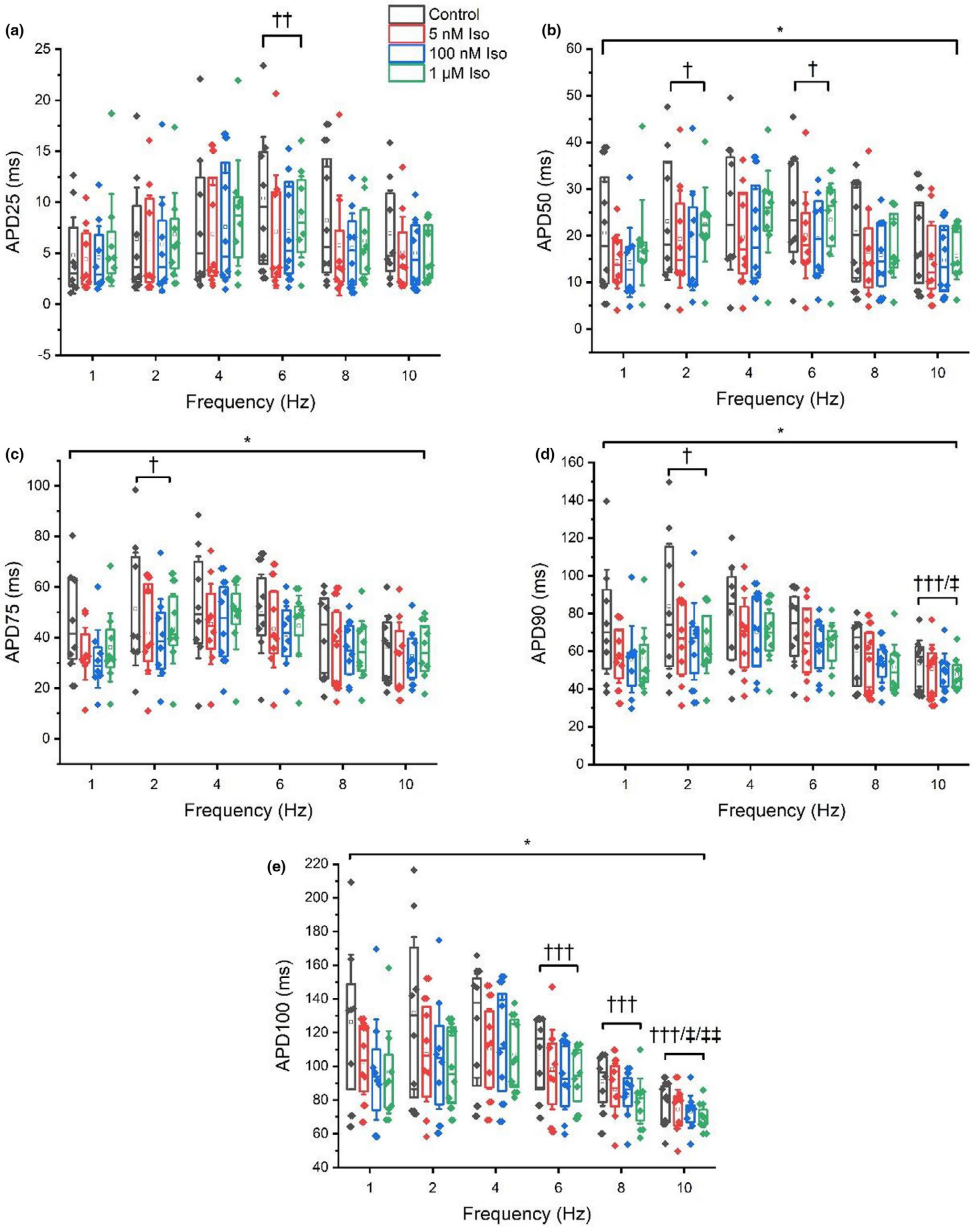
Influence of activation frequency combined with adrenergic stimulation on APD_25_ (a), APD_50_ (b), APD_75_ (c), APD_90_ (d) and APD_100_ (e). * Indicates a significant difference between control and isoproterenol. † Indicates a significant difference compared with 1 Hz. †† indicates a significant difference compared with 2 Hz. ††† Indicates a significant difference compared with 4 Hz. ‡ Indicates a significant difference compared to 6 Hz. ‡‡ Indicates a significant difference compared with 8 Hz. Box plots display mean, median, upper, and lower quartiles and confidence intervals

A repeated measures ANOVA found no statistically significant alterations in APD_25_ between control and adrenergic stimulation (*p* = 0.114). However, APD as measured between 50 and 100% repolarization (APD_50‐100_) showed significant shortening with adrenergic stimulation (*p* =≤ 0.03) (Figures 2, 3), although post‐hoc analyses failed to identify an effect between different doses of isoproterenol.

**FIGURE 4:**
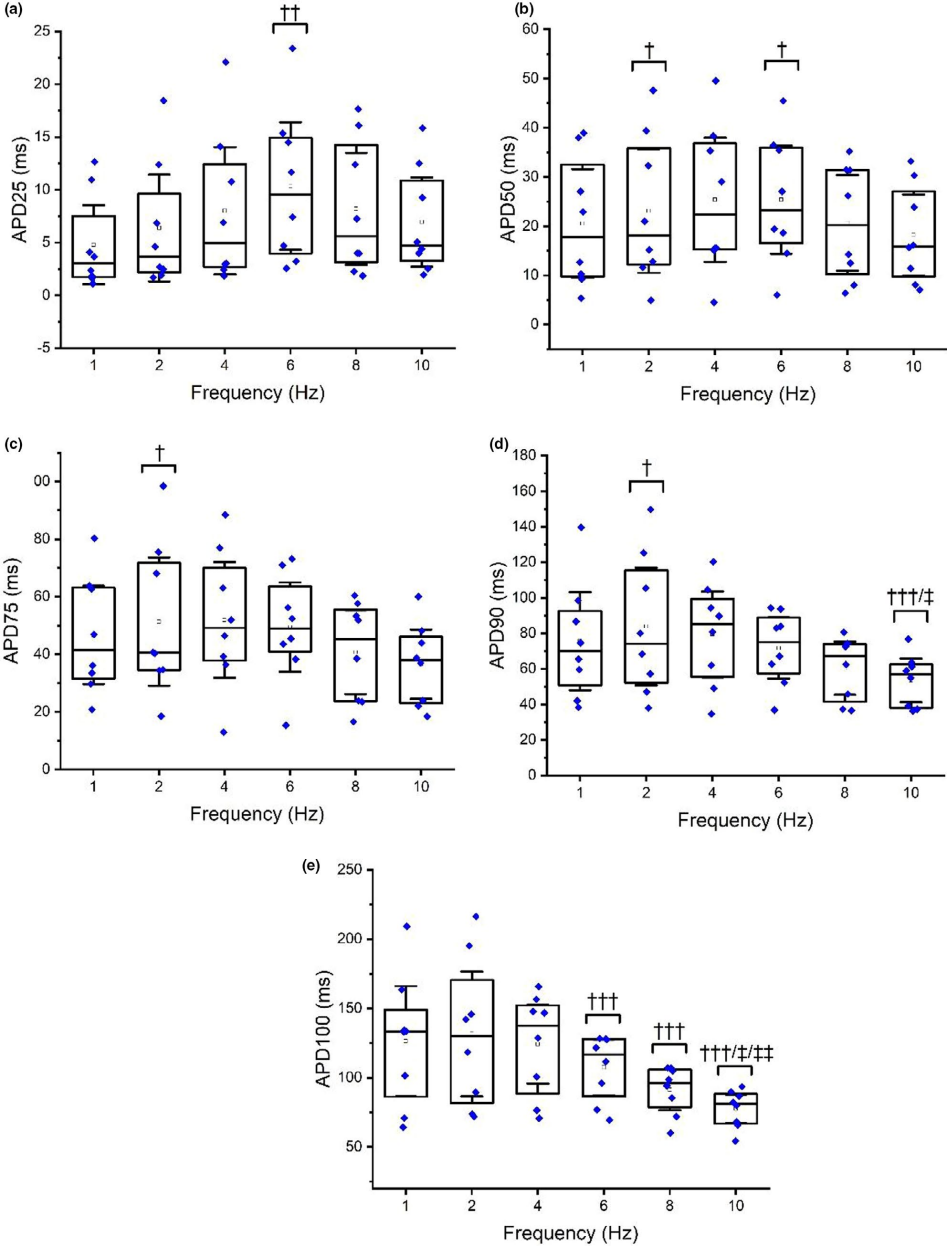
Impact of activation frequency on APD_25_ (a), APD_50_ (b), APD_75_ (c), APD_90_ (d) and APD_100_ (e). † Indicates a significant difference compared with 1 Hz. †† indicates a significant difference compared with 2 Hz. ††† Indicates a significant difference compared to 4 Hz. ‡ Indicates a significant difference compared with 6 Hz. ‡‡ Indicates a significant difference compared with 8 Hz. Box plots display mean, median, upper, and lower quartiles and confidence intervals

Repeated measures ANOVA found APD_25‐100_ were significantly altered by changes in activation frequency (*p* =< 0.03) (Figure 4). Prolongations of APD_25_ were observed at 6 Hz compared to 2 Hz (*p* = 0.02). Prolongations in APD_50_ were observed at 6 Hz and 2 Hz compared to 1 Hz (*p* =< 0.03). Prolongations were also found in APD_75_ at 2 Hz compared to 1 Hz (*p* =< 0.04). Similarly, APD_90_ prolonged at 2 Hz compared to 1 Hz (*p* =< 0.05) and shortened at 10 Hz compared to 4 Hz and 6 Hz (*p* =≤ 0.04). Furthermore, APD_100_ shortened at 10 Hz compared to 4 Hz, 6 Hz and 8 Hz (*p* =< 0.03), whilst APD_100_ also shortened at 6 Hz and 8 Hz compared to 4 Hz (*p* =< 0.04) (Figure 4).

Action potential amplitude was also found to alter significantly during adrenergic stimulation (*p* = 0.013). AP amplitude increased during exposure to 5 nM and 100 nM isoproterenol compared with control at 6 Hz (*p* =< 0.03) (Table 1). Increasing activation frequency however significantly reduced AP amplitude (*p* =< 0.001). Post hoc analyses found AP amplitude was greater at both 1 Hz (*p* = 0.045, *p* = 0.013), and 2 Hz (*p* = 0.025, *p* = 0.02) compared to 4 Hz and 8 Hz (Table 1). Further pairwise comparison found, during control conditions, amplitude was greater at 1 Hz compared to 4 Hz (*p* = 0.029), 6 Hz (*p* = 0.01), 8 Hz (*p* = 0.02) and 10 Hz (*p* = 0.015) (Table 1). During control conditions, amplitude was also greater at 2 Hz compared to 6 Hz (*p* = 0.016) and 8 Hz (*p* = 0.022) and greater at 4 Hz compared to 6 Hz (*p* = 0.011) (Table 1).

**TABLE 1:** Influence of activation frequency and adrenergic stimulation on action potential amplitude. Data presented as mean (± SEM)

	Control		Iso (5 nM)		Iso (100 nM)		Iso (1 μM)	
Frequency (Hz)	Mean (mV)		Mean (mV)		Mean (mV)		Mean (mV)	
1	123.83	(4.25)	113.5	(3.49)	118.7	(3.22)	118.4	(3.62)
2	114.8	(3.69)	112.1	(3.89)	118.2	(3.15)	119.9	(3.06)
4	108.5	(3.29)	110.3	(3.5)	115	(3.41)	116.6	(2.87)
6	100.1	(3.06)	110.4	(2.82)	113.1	(2.84)	115.2	(2.63)
8	98.7	(3.03)	107.6	(3.88)	112.2	(2.41)	113	(2.48)
10	100.6	(2.8)	109.4	(3.33)	113.5	(2.10)	114.3	(2.63)

Diastolic membrane potential became significantly more negative during 100 nM and 1 μM isoproterenol stimulation compared with control conditions (*p* =< 0.03) (Table 2). Pairwise analyses revealed diastolic membrane potential was more negative during 1 µM isoproterenol infusion relative to control at 2 Hz–6 Hz (*p* =< 0.04) (Table 2). At 4 Hz and 6 Hz diastolic membrane potential was less negative under control conditions compared with during 100 nM isoproterenol infusion (*p* =< 0.05). At 8 Hz diastolic membrane potential was less negative under control conditions compared to during 5 nM isoproterenol infusion (*p* = 0.042). At 4 Hz diastolic membrane potential was less negative during 100 nM compared to 1 µM isoproterenol infusion (*p* = 0.038). Finally, at 1 Hz, diastolic membrane potential was more negative at 1 µM compared to 5 nM and 100 nM isoproterenol infusion (*p* =< 0.05) (Table 2). In contrast, no changes were found in diastolic membrane potential with changes in activation frequency (*p* = 0.104), though data in Table 2 shows a trend of diastolic membrane potential becoming more negative at high pacing frequencies compared to low pacing frequencies.

**TABLE 2:** Influence of activation frequency and adrenergic stimulation on diastolic membrane potential. Data presented as mean (± SEM)

	Control		Iso (5 nM)		Iso (100 nM)		Iso (1 μM)	
Frequency (Hz)	Mean (mV)		Mean (mV)		Mean (mV)		Mean (mV)	
1	−68	(2.3)	−70.4	(1.4)	−73.3	(1.7)	−74.8	(1.5)
2	−67.6	(2.5)	−71.5	(1.6)	−73.6	(2.1)	−75.1	(1.9)
4	−67.3	(1.9)	−72.3	(1.9)	−73.3	(1.6)	−75.8	(2)
6	−67.8	(1.4)	−72.8	(1.7)	−74.1	(1.8)	−75.3	(2.2)
8	−70.9	(1.3)	−74.7	(1.5)	−76.2	(2.5)	−76.3	(2.4)
10	−71.9	(2.4)	−74.4	(1.9)	−76	(2.6)	−75.8	(2.4)

Upstroke velocity remained unchanged during adrenergic stimulation (*p* = 0.967) and changes in activation frequency (*p* = 0.067) although there is a trend of slowed upstroke velocity with frequency under control conditions that is not present in saturating doses of isoproterenol (Table 3).

**TABLE 3:** Influence of activation frequency and adrenergic stimulation on upstroke velocity. Data presented as mean (± SEM)

	Control		Iso (5 nM)		Iso (100 nM)		Iso (1 μM)	
Frequency (Hz)	Mean (mV/ms)		Mean (mV/ms)		Mean (mV/ms)		Mean (mV/ms)	
1	154.40	(22.15)	109.90	(13.11)	121.71	(17.00)	103.48	(13.18)
2	124.84	(14.14)	104.63	(13.08)	120.62	(15.99)	110.67	(13.97)
4	108.85	(10.90)	100.99	(11.86)	96.95	(11.52)	104.44	(13.00)
6	84.22	(10.31)	97.62	(10.86)	98.67	(12.00)	104.60	(14.48)
8	82.36	(10.71)	106.18	(12.27)	102.87	(13.52)	101.27	(13.40)
10	95.48	(10.32)	108.95	(9.99)	103.69	(15.06)	116.83	(17.32)

**FIGURE 5:**
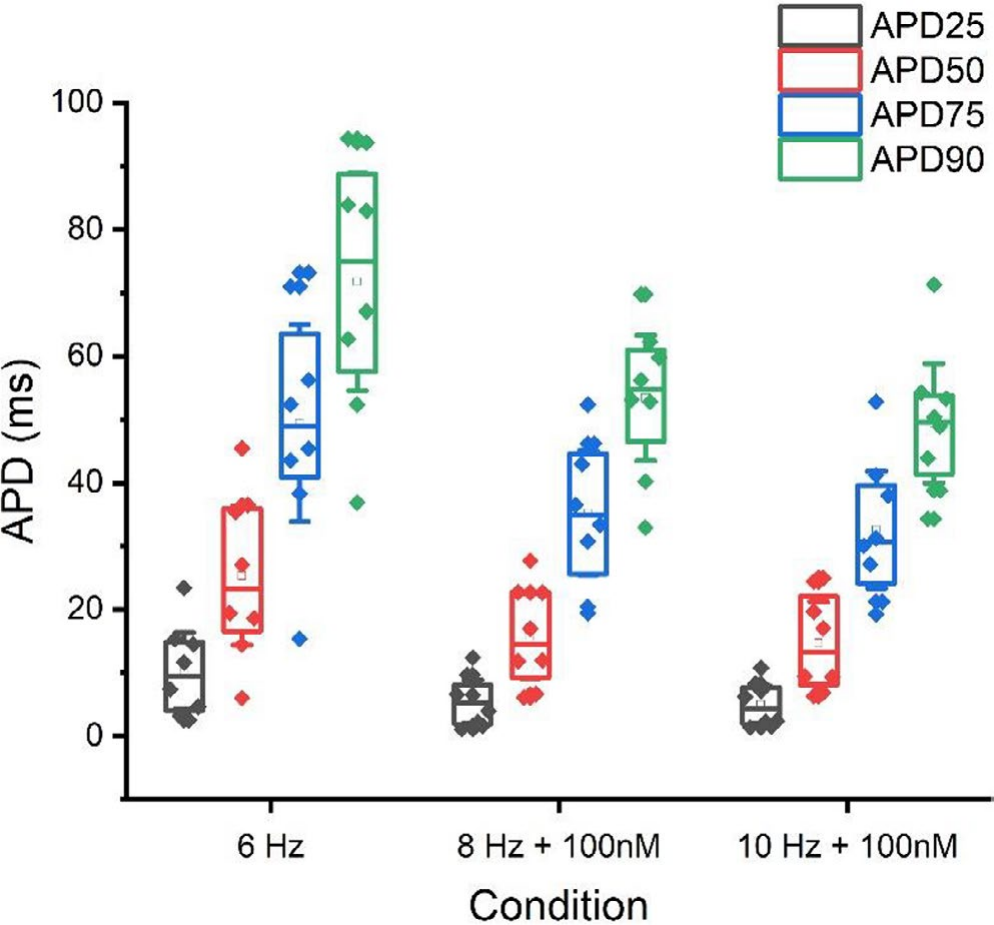
APD response to mimicked exercise in rat ventricular myocytes, with 6 Hz under control conditions emulating physiological resting rat heart rates and 8–10 Hz with added isoproterenol infusion emulating strenuous exercising rat heart rates

**FIGURE 6:**
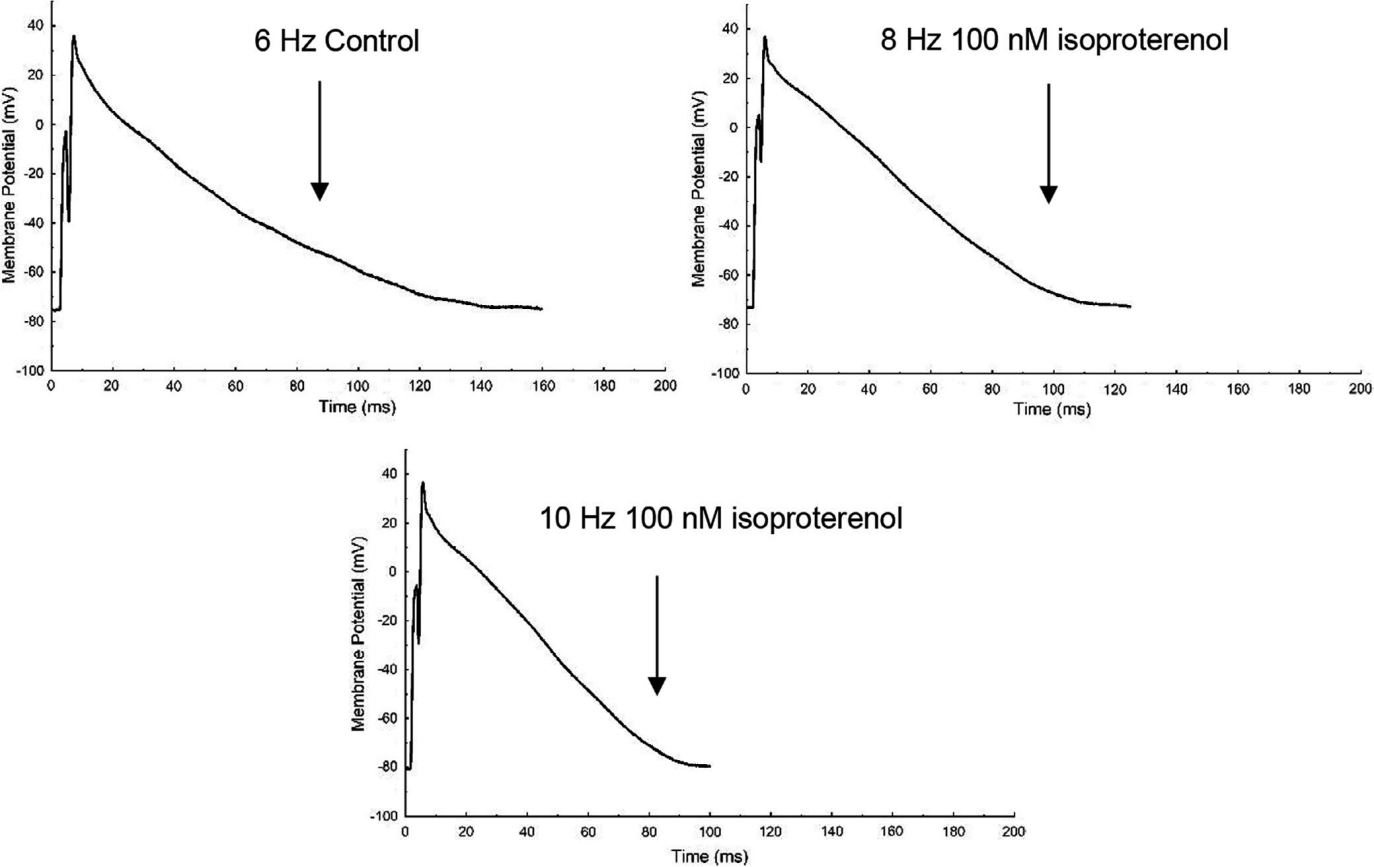
Example action potential response to mimicked exercise in rat ventricular myocytes, with 6 Hz under control conditions emulating physiological resting rat heart rates and 8–10 Hz with added isoproterenol infusion emulating strenuous exercising rat heart rates

Figures 5 and 6 depict the responses in APD_25‐90_ to combined physiologically relevant increments in pacing frequency and adrenergic stimulation found in this study. Such physiological stimuli emulate the rat heart response to typical and more extreme physical activity as might be observed in‐vivo. Specifically, focusing on changes in APD during physiological rest and subsequent strenuous activity in the rat heart help to identify and better understand the real‐time physiologically relevant changes in cellular excitation that will in turn facilitate global contractile modulations in the heart. Figures 5 and 6 show clearly that considerable adaptation occurs during exercise emulation with both early (APD_25_ shortens 46–50% during 100 nM isoproterenol at 8 and 10 Hz pacing respectively compared to 6 Hz pacing during control) and late AP repolarization (APD_75_ and APD_90_ at 8 and 10 Hz pacing shorten 24–29% and 23–28% respectively during 100 nM isoproterenol exposure compared with 6 Hz pacing (control)). The plateau of the AP plateau also shortens (APD_50_ shortens 34–38% during 100 nM isoproterenol at 8 and 10 Hz pacing respectively compared to 6 Hz pacing during control). Equally, the typical variance of the APD reduces as the heart responds to the combined increment in pacing and adrenergic stimulation.

## 4 DISCUSSION

The aim of this study was to assess the influence of activation frequency and adrenergic stimulation on the rat ventricular AP. To our knowledge this is the first study to compare changes in the rat ventricular myocyte AP induced by changes in activation frequency and adrenergic stimulation separately and combined across such a range of stimulation frequencies under conditions designed to replicate normal physiology. Many previous studies in this area have used low activation frequencies (although often resembling those of a human) to investigate cellular electrophysiology or aimed to achieve supra‐maximal saturating doses of adrenergic stimulants, often under room temperature conditions to assess myocyte responses. Such conditions vary considerably from the normal physiology of the model species, potentially impacting experimental outcomes and increasing the difficulty of cross‐study comparisons. Particularly to in‐vivo measures of cardiac function in such animal models. The study provides detailed information regarding AP changes in rate and degree of adrenergic stimulation providing valuable new reference data in particular for higher activation rates and incrementing adrenergic stimulation in ventricular myocytes maintained at body temperature. This will aid the development of computational models, as well as provide reference data for comparison with in‐vivo and intact heart studies.

Our data show that APD_50‐100_ is shortened by submaximal (5 nM), near‐maximal (100 nM) and supra‐maximal (1 μM) adrenergic stimulation irrespective of activation frequency (Figure 3), though no differences were found between individual isoproterenol doses. Furthermore, alterations in activation frequency demonstrate mixed responses in APD. Compared to the commonly used 1 Hz, elevating pacing frequency to more physiological levels relevant to basal rat heart rates (6 Hz) caused prolongations in APD_25‐50_ (Figure 4). Meanwhile, pacing at the highest activation frequencies relevant to intense exercising heart rates in the rat (8 Hz–10 Hz) stimulated significant AP shortening (AP_90‐100_) compared to 1 Hz pacing (Figure 4). Therefore, we are unable to fully accept our initial hypothesis, as increases in activation rate above the typically used 1 Hz frequency did not consistently induce AP shortening, though frequencies above physiological resting rates leads to the shortening of APD. Equally, adrenergic stimulation also stimulated APD shortening, though increments in dose did not lead to clear (statistically significant) additive further shortening.

### 4.1 Influence of adrenergic stimulation on AP repolarization

The shortening of APD_50‐100_ during infusion of isoproterenol found in this study supports previous work (Kang et al., 2017; Stuart et al., 2018; Wang & Fitts, 2017, 2020) and reflects the importance of repolarization reserve and the ability to coordinate APs more efficiently during times of faster stimulation (Figures 2, 3, 5, 6). However, our findings contrast some previous research which found prolongations in APD_90_ of 20% – >50% in response to 10 nM–1 µM isoproterenol (Farrell & Howlett, 2007; Rocchetti et al., 2006; Sala et al., 2017). The significant effect of isoproterenol on APD_50‐100_ coupled with the lack of isoproterenol‐induced changes in AP upstroke velocity indicates the greater role of adrenergic stimulation on AP plateau and AP repolarization in response to physical activity in the ventricles. The lack of statistically significant changes at APD_25_ may be attributed, in part, to the more varied/unpredictable nature of isoproterenol‐induced change (shortening vs prolongation) in early phase repolarization compared to plateau and late repolarization phases (Figure 3).

The lack of statistically significant differences in AP shortening between isoproterenol doses was a surprising finding. The reason for this is not clear. Such findings may suggest that near maximal and supraphysiological doses (100 nM and 1 µM) of adrenergic stimulants elicit effects on myocyte repolarization that are not too dissimilar to the effects of physiological doses (5 nM) when paced at physiologically relevant frequencies. Such an outcome may also be a casualty of the vast heterogeneity of APD results, typical of electrophysiological experiments on global ventricular myocytes. It is also possible that drug application timings maintained some minor influence, whereby, if simply applied for longer, may have caused greater effects. However, lengthening exposure time to progressive doses would have been at the expense of experiment quality and myocyte stability. The time‐periods allowing drug activation in our study were also well established and in line with recent similar studies (Francis Stuart et al., 2018; Wang & Fitts, 2017, 2020). Though too much focus should not be given to isoproterenol dose‐response changes alone (depending on the research question), as adrenergic stimulation does not solely reflect normal physiological behavior during exercise. Instead, greater attention should be applied to the combined effects of rate and adrenergic stimulation, ideally during conditions physiologically relevant to the in‐vivo cardiac response to exercise, in which this study found the elevation of activation frequency above physiological resting rat heart rates along with the infusion of isoproterenol resulted in significant APD shortening (Figures 2, 3, 5, 6). Such findings potentially illustrate the impact of underlying modulations in calcium and K^+^ current balances in the heart during moderate to strenuous exercise such as reducing L‐type calcium current inactivation time and increasing transient outward, slow and rapid delayed rectifying K^+^ currents and inward rectifying K^+^ currents.

Furthermore, the percentage change of APD shortening induced by adrenergic stimulation during later repolarization (APD_75‐100_) showed a trend of dampening at the highest frequencies such as 8 Hz and 10 Hz pacing compared to lower frequencies such as 1 Hz and 2 Hz pacing (Figures 2, 3). An opposite trend was observed of early repolarization, as APD_25_ prolonged at low frequencies (1, 2 and 4 Hz) and shortened at higher frequencies (6, 8 and 10 Hz) relative to control (Figure 3). The findings in later repolarization might be expected as the restitution of ionic currents to allow high frequencies to be followed requires enhanced recruitment of repolarization currents. Little data on isoproterenol‐induced changes at higher frequency activation exists to provide comparison to our findings, however a body of evidence in various species exists regarding isoproterenol responses to low frequency activation (Kang et al., 2017; Rocchetti et al., 2006; Sala et al., 2017; Stuart et al., 2018; Wang & Fitts, 2017). Existing literature predominantly demonstrates isoproterenol (10 nM to 1 µM) induces APD_80‐90_ shortening in magnitudes ranging between 7 and 43% among mice, humans, dogs, and rats at low frequencies (0.5–2 Hz) (Kang et al., 2017; Sala et al., 2017; Stuart et al., 2018; Wang & Fitts, 2017). Such data correlates well with the findings of this study with isoproterenol stimulating reductions in APD_75‐90_ during 1 Hz (APD_75_: 25 ± 4%, APD_90_: 21 ± 3%) and 2 Hz (APD_75_: 13 ± 6%, APD_90_: 15 ± 5%) activation frequencies.

Interestingly, previous literature suggests rats operate a mixed force‐frequency relationship relative to other mammals (Boyet & Jewell, 1981; Carmeliet, 2006). This increases the difficulty of making robust comparisons along varied levels of stimulation. It is generally understood that most mammals exhibit stepwise APD reductions as pacing frequency increases (Boyet & Jewell, 1981; Carmeliet, 2006). In rat models however, controversy exists, as APDs may equally shorten or become prolonged (depending on experimental conditions and solutions) at greater frequencies (Boyet & Jewell, 1981; Carmeliet, 2006), as also evidenced in this study, though a relatively narrow pacing range has been used previously in the literature. It is likely that this non‐uniformity in APD response to activation frequency yields consequences for responses during adrenergic stimulation and may also have contributed to the lack of significant further APD changes induced by adrenergic stimulation between individual doses and activation frequencies involved in this study.

### 4.2 Influence of activation frequency on AP repolarization

The shortening of APD_90‐100_ at the highest pacing frequencies was expected and supports some previous findings (Wang & Fitts, 2017). At 8 Hz–10 Hz, which is (partially) reflective of heavy exercise in a rat model, APD must shorten to satisfy the declining diastolic interval and demonstrates the use of repolarization reserve. In the absence of isoproterenol this study demonstrated shortening of APD_90‐100_ at 10 Hz compared to 6 Hz, potentially reflecting (in part) the above‐mentioned cardiac acceleration between physiological rest and strenuous physiological exercise in the rat by magnitudes of 25–28% (Figure 4).

The APD_50_ prolongation occurring at 2 Hz and 6 Hz frequencies compared to the initial and most frequently quoted 1 Hz frequency irrespective of adrenergic stimulation, is particularly interesting. The logical progression from a pacing frequency of 1 Hz for APD would in theory be to reduce, demonstrating a stepwise/linear APD‐rate relationship (Liu et al., 1993), though in the absence of isoproterenol, this study reported prolongations in APD_25_ at 6 Hz compared to 2 Hz and APD_50_ at 2 and 6 Hz pacing compared to 1 Hz. This is contrary to similar research which has found reductions in APD_20‐50_ of approximately 43% with increases in activation frequency (2.5–4 Hz) in rats, though this quoted study was performed in atrial rather than ventricular myocytes (Huang et al., 2006). The underlying mechanism responsible for such findings is unclear. It is likely ion fluxes influencing repolarization, particularly calcium and potassium channels, vary considerably with alterations in pacing frequency and alter the balance between AP shortening and prolongation. The results of this study appear to suggest rate‐dependent changes in AP repolarization primarily involve modulation of the late phase repolarization at the highest activation frequencies, whilst rate‐dependent changes at lower activation frequencies involve early repolarization and plateau phases (Figure 4). It may also be that, at these intermediate pacing frequencies (4 Hz–6 Hz), myocytes are functioning at approximate or near‐approximate basal rates and are physiologically predisposed to slower APDs early in the AP contour in comparison to the artificial pacing rate of 1 Hz. Such mixed APD responses to changes in activation frequency have been alluded to previously in research and further supports the nonuniformity in the force‐frequency relationship / APD rate‐dependency in rats as mentioned above (Boyet & Jewell, 1981; Carmeliet, 2006).

### 4.3 Influence of varied stress on AP amplitude and diastolic membrane potential

Alterations in AP amplitude during adrenergic stimulation ranged from an 8% reduction to a 15% increase with significant increases in AP amplitude occurring during 5 nM (10 ± 2%) and 100 nM (13 ± 4%) isoproterenol infusion compared to control at 6 Hz. Meanwhile, AP amplitude measured in the absence of isoproterenol appeared to demonstrate a decline as activation frequency was increased from 1 Hz through to 10 Hz (Table 1). However, Table 1 displays at activation frequencies of 4 Hz and greater, the infusion of isoproterenol appeared to restore the frequency‐related losses in amplitude to some extent. Previous research has also found reductions in AP amplitude with increases in rate, however the underlying mechanism is unclear (Ravens & Wettwer, 1998). Similarly, our findings on AP amplitude in response to adrenergic stimulation support previous research and show modest increases (Verkerk et al., 2012; Wang et al., 2009). This is typically thought to be a result of changes in sodium current, which has been found to increase with isoproterenol infusion and is associated with upregulations of conduction velocity, beneficial during periods of increased stress (Wang et al., 2009).

The lack of changes in upstroke velocity in our study is in line with previous literature which paints a mixed picture of modulations in upstroke velocity when subject to stress, particularly in the form of adrenergic stimulation (King et al., 2013; Liu et al., 1999; Verkerk et al., 2012). The lack of changes in upstroke velocity may be indicative of the greater importance of phase 2 and 3 of the AP contour compared to phase 0 in augmenting myocyte function during β_1_AR signaling.

This study demonstrated diastolic membrane potential was not changed by activation frequency (despite a trend toward more negative potentials at higher pacing frequencies) but became 9 ± 1% and 11 ± 1% more negative during 100 nM and 1 μM isoproterenol infusion compared to control irrespective of activation frequency. A similar trend was observed during infusion of a more physiological dose of isoproterenol (5 nM), which displayed diastolic membrane potential became 6 ± 1% more negative irrespective of activation frequency, though this was not significant. These results support previous evidence which suggests diastolic membrane potential undergoes hyperpolarization during adrenergic stimulation potentially through increased potassium channel conductance, though isoproterenol has been shown to both elevate and decrease the main rectifying potassium current (Chiamvimonvat et al., 2017; Crumbie, 2016; Gadsby, 1983; Verkerk et al., 2012).

## 5 PRACTICAL APPLICATIONS

The findings of this study provide an insight to the influence of varied stress on repolarization and generate important reference values for further study regarding a whole range of physiological frequencies as well as physiological and saturating doses of isoproterenol previously not studied. The varied findings in this study also highlight the importance of replicating physiologically relevant environments and exploring varied stress during patch‐clamp investigations. This data can be utilized for the equally important development and validation of computational models.

## 6 LIMITATIONS

Every effort was made during this study to minimize limitations, however inevitably some remained. The results of this study apply to 3‐month‐old male rats, significantly different outcomes might be expected in rats of a different age, gender and health status due to recognized physiological differences. As an additional consideration all myocyte recordings were made from a small, randomized sample of myocytes taken from the complete collected pool of ventricular myocytes isolated from each heart. The documented heterogeneity of APD across ventricular regions has not been considered in this study and further work is required to investigate if the responses are common to all sub‐regions. As a further consideration for the application of this work to in‐vivo physiology, whilst the APD measured under control conditions are comparable to those measured using less‐intrusive approaches such as mono‐phasic APs from the epicardial surface, the intrusive nature of patch‐clamp recordings will potentially influence the observed data. Patch clamp recordings by their nature disrupt the intracellular milieu and can potentially influence activation and inactivation of ion channels due to intracellular dilution and disruption of ion buffering. The patch solution in these experiments had a typical high potassium basis as is generally used to match expected intracellular conditions, but to promote stability during the experiments also contained EGTA as a calcium buffer. The disruption of normal calcium buffering is common to many experiments on myocytes, whether using cellular indicators or for preserving cellular integrity such as in these experiments, but does have the potential to influence numerous calcium‐dependent processes that could influence the AP. Whilst the APDs we report are comparable to some recorded from the epicardial surface as monophasic APs, a detailed investigation of the potential influence of changes in calcium buffering on the AP response would further address questions regarding the role this may play in influencing the adrenergic response as well as the rate‐dependency of the APs. Some previous work shows that introduction of a faster calcium buffer such as BAPTA did not influence early phases of the AP but did influence later phases to cause a degree of shortening under conditions resembling the control conditions of our experiments (Shipsey et al., 1997) but the influence on overall response to rate and isoproterenol is not known.

## 7 CONCLUSION

This study yielded interesting findings in relation to an initial rate‐dependent prolongation of early phase repolarization and AP plateau, subsequently followed by shortening of AP plateau and late phase repolarization, alongside a lack of clear dose‐related APD shortening with increasing adrenergic stimulation. These results demonstrate the varied effect of adrenergic stimulation and activation frequency on myocyte repolarization and contributes to novel APD reference values in a rat model under varied physiological stress. Such mixed effects may be explained by alterations in ion channels modulating AP repolarization and potentially by the inherent lack of uniform rate‐dependent AP modulation documented in the rat model. Further research identifying the ion channel flux changes responsible for both frequency and adrenergic AP responses is required. The findings of this study indicate the potential need for a greater focus on ion channels responsible specifically for the AP plateau and late repolarization phases when investigating the modulation of repolarization during adrenergic stimulation. This work provides the foundations for potential future comparisons across differing genders, ages, and disease statuses where sensitivity and response to adrenergic stimulation and frequency may alter. The data yielded from this study will also contribute to reliably modelling rat heart responses to exercise using computational techniques.

## CONFLICT OF INTEREST

There were no personal, professional, or financial conflicts influencing this study.

## AUTHOR CONTRIBUTIONS

Author contributions LH: conception; study design; assisting myocyte isolation; electrophysiological data collection; data analysis; data interpretation; drafting of the original manuscript. HK: animal sacrifice; myocyte isolation; myocyte isolation training; critical revision of manuscript. MA: assisting myocyte isolation; critical revision of manuscript. DS: animal acquisition; critical revision of manuscript. ML: conception; study design; myocyte isolation training; electrophysiology training; data interpretation; critical revision of manuscript.
